# Validity and reliability of a novel iPhone method to rapidly measure cervical sagittal parameters

**DOI:** 10.1038/s41598-022-21660-z

**Published:** 2022-11-15

**Authors:** Jie Zhang, Chunyang Zhang, Weiyang Zhong, Zenghui Zhao, Fei Han, Zhenghan Han, Hang Zhang, Tianji Huang, Xiaoji Luo

**Affiliations:** 1 Department of Oncology, The First Affiliated Hospital of Chongqing Medical University, Chongqing, 400016 People’s Republic of China; 2 Department of Orthopedic Surgery, People’s Hospital of Chongqing Banan District, Chongqing, 401320 People’s Republic of China; 3 Department of Orthopedic Surgery, The First Affiliated Hospital of Chongqing Medical University, Chongqing, 400016 People’s Republic of China; 4 Orthopedic Laboratory of Chongqing Medical University, Chongqing, 400016 People’s Republic of China

**Keywords:** Bone imaging, Radiography, Orthopaedics, Musculoskeletal system, Bone, Skeleton

## Abstract

We introduced a novel method based on the iPhone’s intrinsic photo edit function to measure sagittal parameters of the cervical spine. This study aimed to assess the validity of this new method compared with the picture archiving and communication system (PACS) method (the gold standard) and to test the reliability of this novel technique. One hundred consecutive patients admitted to our hospital diagnosed with cervical spondylotic myelopathy or cervical spondylotic radiculopathy were retrospectively reviewed. Four angles, including the C0-2 Cobb angle, C2-7 Cobb angle, T1S and neck tilt (NT), were assessed by iPhone and PACS. The validity and reliability were evaluated, and the time taken by both methods was compared. The ICCs of the validity of the C0-2 Cobb angle, C2-7 Cobb angle, T1S and NT were 0.960, 0.976, 0.980 and 0.946, respectively. The ICCs of the intraobserver reliability of the C0-2 Cobb angle, C2-7 Cobb angle, T1S and NT were 0.966, 0.983, 0.971 and 0.951, respectively. The ICCs of the interobserver reliability of the C0-2 Cobb angle, C2-7 Cobb angle, T1S and NT were 0.953, 0.972, 0.957 and 0.929, respectively. The Bland‒Altman plot of validity of the four angles revealed mean differences of 0.3, 0.2, 0.1, and 0.1 degrees with 95% CIs of 4.1, 4.1, 2.9, and 4.3 degrees, respectively. The iPhone measurement time (58.55 ± 4.17 s) was significantly less than that by the PACS (70.40 ± 2.92 s) when compared by the independent-samples T test (*P* < 0.001). This novel method using the iPhone’s intrinsic photo edit function is accurate, reliable, fast and convenient when measuring cervical sagittal parameters.

## Introduction

There have been a growing number of studies concerning the radiographic characteristics and associated clinical outcomes regarding cervical sagittal parameters^1^, which may be used to evaluate the prognosis and clinical effects of many cervical disorders, such as tuberculosis^2^, ossification in the posterior longitudinal ligament and cervical spondylosis myelopathy^3^, as well as surgical assessments, including laminoplasty^3^, anterior cervical discectomy and fusion^4^ and cervical disc arthroplasty^5^. The C0-2 Cobb angle can be used to assess the level of surgical invasion to the posterior muscular-ligament complex after open-door laminoplasty^6^. In patients with cervical spondylotic radiculopathy associated with a single segment, a C2-7 Cobb angle of more than 7.7° suggests a greater possibility of effectiveness of conservative treatment^7^. In cervical myelopathy patients who receive laminoplasty, the T1-slope (T1S) has been suggested to indicate a change in kyphotic alignment^8^. Studies have revealed that NT remains stable after surgical correction of kyphosis, suggesting that NT plays a role in the balance of the cervical spine and head^9^. Therefore, it is necessary to find a method that can measure cervical sagittal parameters accurately, quickly and conveniently. Shortcomings have been noticed with respect to traditional measurements with marker pens and protractors, the picture archiving and communication system (PACS) method as the gold standard, and smartphone methods with special apps developed in recent years^10^. Therefore, we introduced a novel method based on the iPhone’s intrinsic photo edit function to measure sagittal parameters of the cervical spine. This study aimed to assess the validity of this new method compared with the PACS method (the gold standard) and to test the reliability of this novel method. The time taken by these two methods was also compared.

## Materials and methods

All methods were carried out in accordance with relevant guidelines and regulations. This study was approved, and the requirement for informed consent was waived by the ethics committee of The First Affiliated Hospital of Chongqing Medical University.

### Patient selection

One hundred consecutive patients admitted to our hospital diagnosed with cervical spondylotic myelopathy or cervical spondylotic radiculopathy were retrospectively reviewed. The inclusion criteria were legible lateral plain films of the cervical spine. The exclusion criteria were kyphosis of the cervical spine.

### Measurement methods

**Figure 1:**
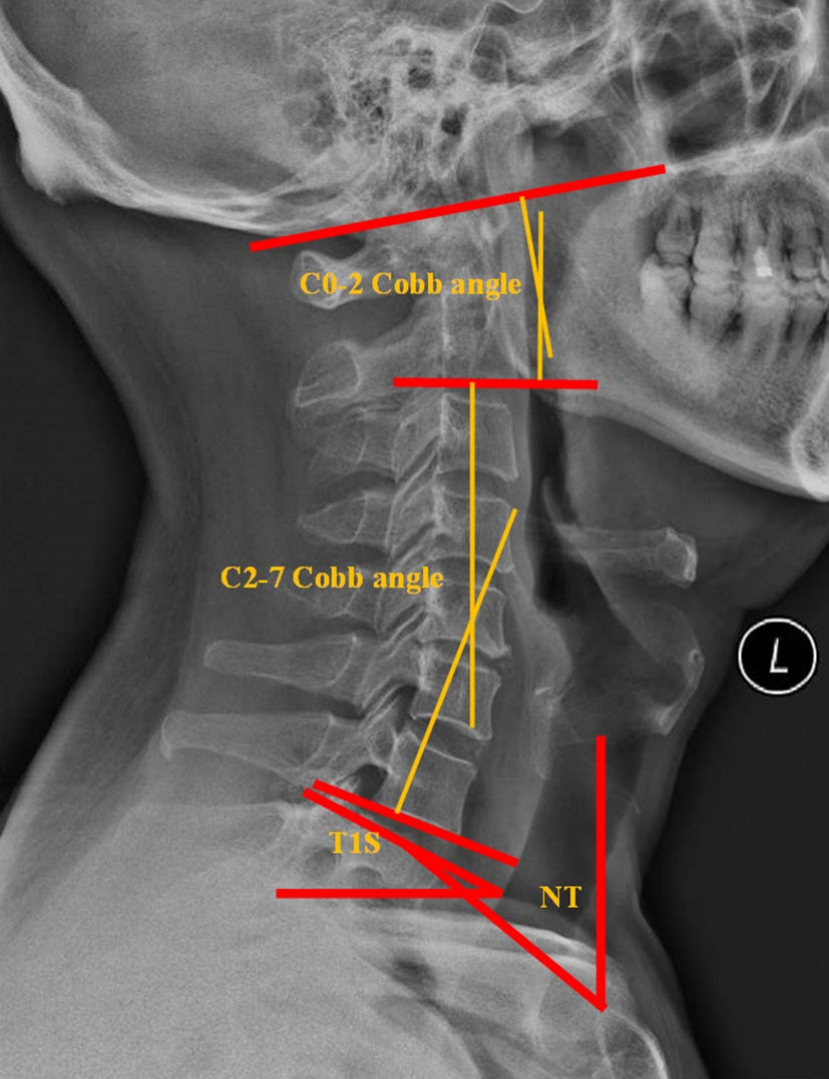
The definition of the C0-2 Cobb angle, C2-7 Cobb angle, T1S and NT.

Four angles, including the C0-2 Cobb angle, C2-7 Cobb angle, T1S and neck tilt (NT), were chosen to represent the sagittal parameters of the cervical spine in the present research. The C0-2 Cobb angle was defined as the included angle between the McGregor line and the line parallel to the C2 lower endplate (or the included angle that lines perpendicular to the McGregor line and the C2 lower endplate); the C2-7 Cobb angle was defined as the included angle between the line parallel to the C2 lower endplate and the line parallel to the C7 lower endplate (or the included angle that lines perpendicular to the C2 lower endplate and the C7 lower endplate); T1S was defined as the included angle between the T1 upper endplate and horizontal line; NT was defined as the included angle between the vertical line and the line connecting the upper end of the sternum and the centre of the T1 upper endplate^3,8,11,12^ (Fig. 1).

To minimize the measurement deviation, the same computer screen and the same iPhone were used in this study. PACS’s intrinsic system was used to manually measure cervical sagittal parameters by marking the lines and reading them automatically.

**Figure 2:**
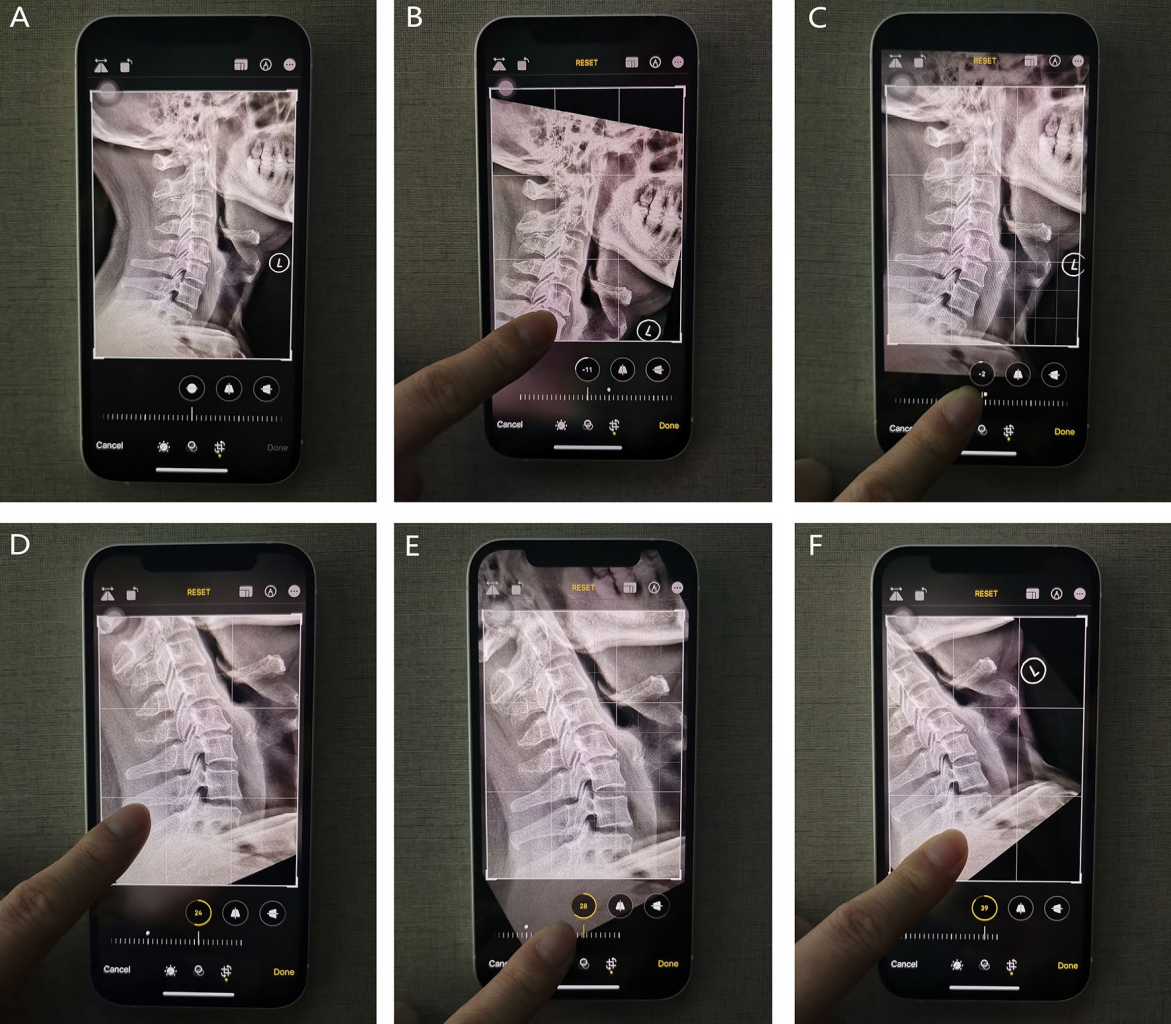
Measurement of the C0-2 Cobb angle, C2-7 Cobb angle, T1S and NT by the iPhone’s intrinsic photo edit function. (**A)** The targeted photo was selected to be edited. (**B**) The photo was rotated until the McGregor line overlapped or was parallel to the horizontal grid lines, and then this angle was recorded. (**C**) The photo was rotated until the lower endplate of C2 overlapped or was parallel to the horizontal grid lines, and then this angle was recorded. (**D**) The photo was rotated until the lower endplate of C7 overlapped or was parallel to the horizontal grid lines, and then this angle was recorded. (**E**) The photo was rotated until the upper endplate of T1 overlapped or was parallel to the horizontal grid lines, and this angle was recorded. (**F**) The photo was rotated until the line connecting the sternum’s upper end and the centre of the T1 upper endplate was overlapping or parallel to the horizontal grid lines, and this angle was recorded.

The built-in camera of the iPhone was used to take photos for measurement. To minimize parallax errors, the iPhone screen’s plane should be kept parallel to the plane of the computer screen when taking the photo. The built-in photo edit function was used to rotate the pictures of the X-ray films, allowing the grid lines and scale of rotation angle to be identified clearly and simultaneously (Fig. 2A–E).

To measure the C0-2 Cobb angle, first, the photo was rotated until the McGregor line was overlapping or parallel to the horizontal grid lines, and then this angle was recorded; second, the photo was rotated until the lower endplate of C2 was overlapping or parallel to the horizontal grid lines, and then this angle was recorded; finally, the difference between these two angles that represented the C0-2 Cobb angle was calculated and recorded (Fig. 2B,C).

To measure the C2-7 Cobb angle, first, the photo was rotated until the lower endplate of C2 was overlapping or parallel to the horizontal grid lines, and then this angle was recorded; second, the photo was rotated until the lower endplate of C7 was overlapping or parallel to the horizontal grid lines, and then this angle was recorded; finally, the difference between these two angles that represented the C2-7 Cobb angle was calculated and recorded (Fig. 2C,D).

To measure T1S, the photo was rotated until the upper endplate of T1 was overlapping or parallel to the horizontal grid lines, and this angle was recorded as T1S (because the horizontal line always represented 0°) (Fig. 2E).

To measure NT, the photo was rotated until the line connecting the upper end of the sternum and the centre of the T1 upper endplate overlapped or was parallel to the horizontal grid lines, and this angle was recorded. NT was the supplementary angle of this angle (if NT was less than 45°, the photo was rotated until the line connecting the upper end of the sternum and the centre of the T1 upper endplate overlapped or was parallel to the vertical grid lines, and this angle was recorded as the NT (Fig. 2F).

One attending spine surgeon as the experienced observer (observer A) and one orthopaedic resident in training as the inexperienced observer (observer B) independently reviewed the radiographs. The observers were blinded to the patient information. Observer A and observer B both took photos from the iPhone and measured the angles independently. To evaluate the interobserver reliability, the cervical sagittal parameters were measured with the iPhone’s intrinsic photo edit function by two of the observers. Observer A measured the angles with the iPhone again to evaluate the intraobserver reliability and with the PACS to assess the validity of the novel iPhone method^13^. The interval between each round of measurements was more than two weeks. The order of the radiographs was reorganized randomly to decrease possible recall. A stopwatch was used to record the time of each measurement. To simulate the actual measurement process, the time of measurement by the PACS began with the input of the radiograph number into the PACS and ended with the recording of the angle. The time of measurement of the iPhone began with the turning on of the camera function and ended with the recording of the angle. Excel 2016 was used to record the angle data.

### Statistical analysis

All the data were analysed by SPSS 21.0 blinded. Intraclass correlation coefficients (ICCs) were used to evaluate the validity and the intraobserver and interobserver reliability. The validity of this novel iPhone method was assessed by the PACS measurement taken by observer A and the mean outcomes of two iPhone measurements taken by observer A. The intraobserver reliability was assessed by comparing the outcomes of two measurements by observer A with the iPhone. The interobserver reliability was evaluated by comparing the outcomes between the mean outcomes of two iPhone measurements taken by observer A and the only measurement taken by observer B^13^.

A good ICC is defined as over 0.75, a very good ICC is defined as over 0.85, and an excellent ICC is defined as over 0.9^13^. A Bland‒Altman plot, with the mean difference and 95% confidence interval (CI) visualized by MedCalc software, was used to graphically evaluate the validity and intra- and interobserver reliability of this novel method. The time difference of the PACS and iPhone methods was assessed by the times taken by the PACS and the mean time of two measurements of the iPhone taken by observer A, which was conducted by an independent-samples T test.

## Results

### General information

This study reviewed 100 lateral plain films from cervical spondylotic radiculopathy or cervical spondylotic myelopathy patients, including 45 males and 55 females. The average age was 57.14 ± 11.05 years old. The C0-2 Cobb angle measured by the PACS was 21.85 ± 7.62°, and the mean C0-2 Cobb angle measured by the iPhone was 21.46 ± 7.24°. The C2-7 Cobb angle measured by the PACS was 15.39 ± 9.19°, and the mean C2-7 Cobb angle measured by the iPhone was 15.69 ± 9.51°. The result of T1S measured by the PACS was 24.03 ± 7.40°, and the mean result of T1S measured by the iPhone was 24.04 ± 7.52°. The NT measured by the PACS was 52.11 ± 6.57°, and the mean NT measured by the iPhone was 52.13 ± 6.77°. The measuring time by iPhone’s intrinsic photo edit function (58.55 ± 4.17 s) was significantly less than that by the PACS (70.40 ± 2.92 s) when compared by independent-samples T test (*P* < 0.001).

### Validity

**Figure 3:**
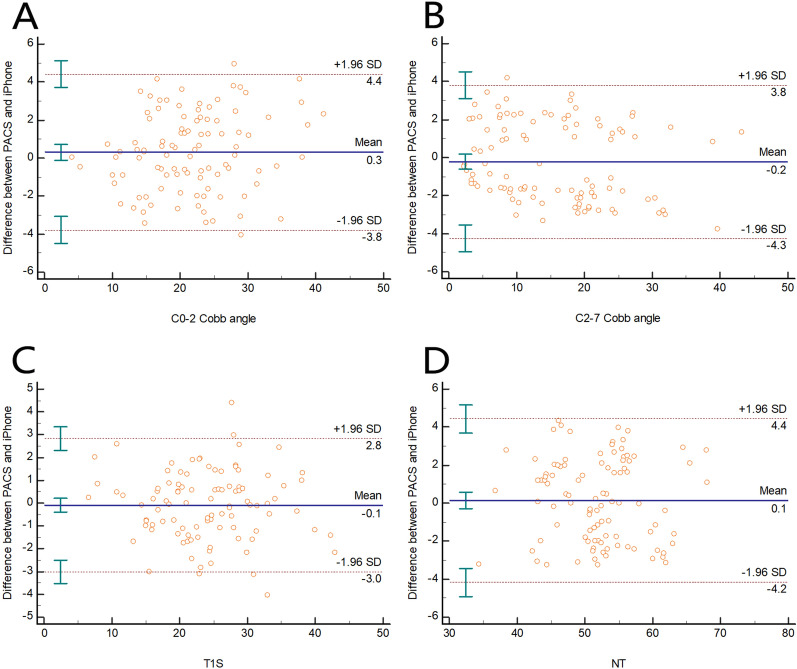
Bland‒Altman plot revealing the difference between the PACS and iPhone measurements for the (**A**) C0-2 Cobb angle, (**B**) C2-7 Cobb angle, (**C**) T1S, and (**D**) NT. The mean difference is shown as the solid lines in the middle, and the 95% CI is presented as the outer two dotted lines.

The ICC of validity of the C0-2 Cobb angle was 0.960 (0.941–0.973), and the Bland‒Altman plot revealed that the 95% CI was 4.1°, while the mean difference was 0.3° (Fig. 3A). The ICC of validity of the C2-7 Cobb angle was 0.976 (0.965–0.984), and the Bland‒Altman plot revealed that the 95% CI was 4.1°, while the mean difference was 0.2° (Fig. 3B). The ICC of validity of the T1S was 0.980 (0.970–0.986), and the Bland‒Altman plot revealed that the 95% CI was 2.9°, while the mean difference was 0.1° (Fig. 3C). The ICC of validity of NT was 0.946 (0.921–0.963), and the Bland‒Altman plot revealed that the 95% CI was 4.3°, while the mean difference was 0.1° (Fig. 3D) (Table 1).

**Table 1:** The ICC, 95% limits of agreement (LoA) and mean absolute percentage error (MAPE) of validity, intraobserver and interobserver reliability of the four angles.

	Validity	Intraobserver reliability	Interobserver reliability
**C0-2 Cobb angle**			
ICC	0.96	0.966	0.953
95% LoA	(− 3.8 to + 4.4)	(− 3.6 to + 3.9)	(− 4.1 to + 4.6)
MAPE	8.40%	8.00%	9.90%
**C2-7 Cobb angle**			
ICC	0.976	0.983	0.972
95% LoA	(− 4.3 to + 3.8)	(− 3.5 to + 3.5)	(− 4.6 to + 4.1)
MAPE	17.00%	14.70%	21.20%
**T1S**			
ICC	0.98	0.971	0.957
95% LoA	(− 3.0 to + 2.8)	(− 3.3 to + 3.7)	(− 4.0 to + 4.6)
MAPE	5.40%	7.10%	7.90%
**NT**			
ICC	0.946	0.951	0.929
95% LoA	(− 4.2 to + 4.4)	(− 4.2 to + 4.2)	(− 5.3 to + 4.5)
MAPE	3.70%	3.60%	4.60%

### Intraobserver reliability

**Figure 4:**
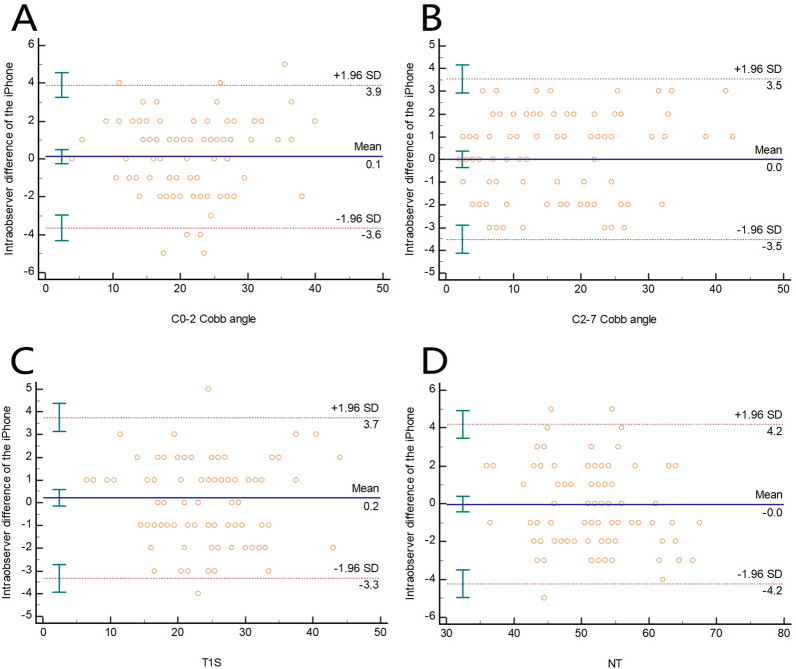
Bland‒Altman plot revealing the intraobserver difference of the iPhone measurement for the (**A**) C0-2 Cobb angle, (**B**) C2-7 Cobb angle, (**C**) T1S, and (**D**) NT. The mean difference is shown as the solid lines in the middle, and the 95% CI is presented as the outer two dotted lines.

The ICC of the intraobserver reliability of the C0-2 Cobb angle was 0.966 (0.950–0.977), and the Bland‒Altman plot revealed that the 95% CI was 3.8°, while the mean difference was 0.1° (Fig. 4A). The ICC of the intraobserver reliability of the C2-7 Cobb angle was 0.983 (0.974–0.988), and the Bland‒Altman plot revealed that the 95% CI was 3.5°, while the mean difference was 0.0° (Fig. 4B). The ICC of intraobserver reliability of T1S was 0.971 (0.958–0.981), and the Bland‒Altman plot revealed that the 95% CI was 3.5°, while the mean difference was 0.2° (Fig. 4C). The ICC of intraobserver reliability of NT was 0.951 (0.928–0.967), and the Bland‒Altman plot revealed that the 95% CI was 4.2°, while the mean difference was 0.0° (Fig. 4D) (Table 1).

### Interobserver reliability

**Figure 5:**
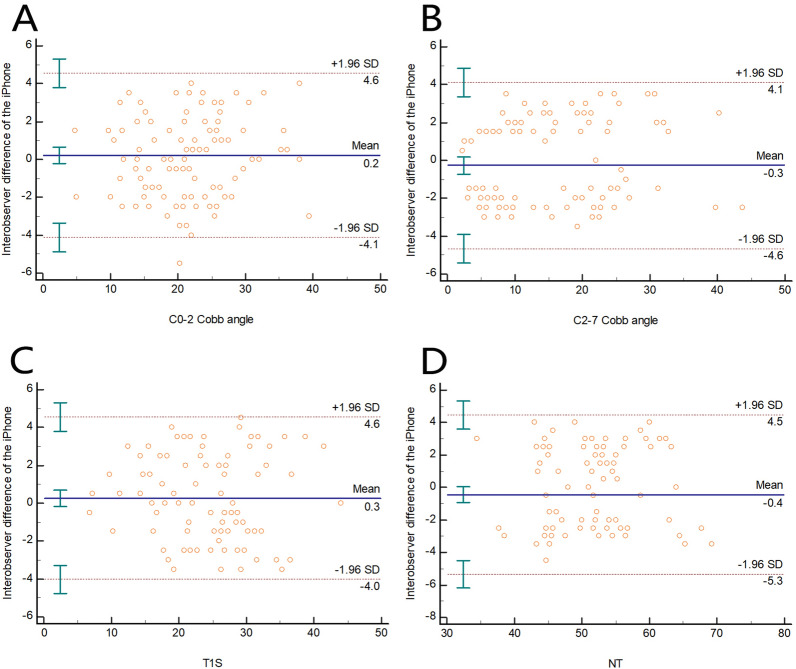
Bland‒Altman plot revealing the interobserver difference of the iPhone measurement for the (**A**) C0-2 Cobb angle, (**B**) C2-7 Cobb angle, (**C**) T1S, (**D**) NT. The mean difference is shown as the solid lines in the middle, and the 95% CI is presented as the outer two dotted lines.

The ICC of interobserver reliability of the C0-2 Cobb angle was 0.953 (0.930–0.968), and the Bland‒Altman plot revealed that the 95% CI was 4.4°, while the mean difference was 0.2° (Fig. 5A). The ICC of interobserver reliability of the C2-7 Cobb angle was 0.972 (0.959–0.981), and the Bland‒Altman plot revealed that the 95% CI was 4.4°, while the mean difference was 0.3° (Fig. 5B). The ICC of interobserver reliability of T1S was 0.957 (0.937–0.971), and the Bland‒Altman plot revealed that the 95% CI was 4.3°, while the mean difference was 0.3° (Fig. 5C). The ICC of interobserver reliability of NT was 0.929 (0.897–0.952), and the Bland‒Altman plot revealed that the 95% CI was 4.9°, while the mean difference was 0.4° (Fig. 5D) (Table 1).

## Discussion

Traditionally, cervical sagittal parameters are measured by drawing lines on the radiographic films with a marker pen and reading them with a protractor. These tools are not always carried by doctors, and stained film and time-consuming issues are concerns that should also be noted. As the current gold standard, the PACS method can measure the parameters quickly and accurately. However, the feature of being based on the computer system in a hospital renders this method not portable, with no social function. Moreover, the PACS is not always compatible among different hospitals, and the radiographs of one hospital cannot always be measured in the system of other hospitals^14^. With the popularity of electronic products, some iPhone apps have been developed to assess orthopaedic parameters. However, defects of these apps, such as download fees, less stability compared to that of the intrinsic function of smartphones, lack of updates, and lack of applicability to smartphone systems other than the iPhone system, cannot be ignored^10^.

Therefore, in the present research, we introduced a new measuring method based on the iPhone’s intrinsic photo edit function to measure cervical sagittal parameters. This novel method has proven useful in the measurement of lumbar pelvic parameters and hallux valgus parameters^10,14^. In the present study, the validity of this new method with the PACS method was compared, and the intraobserver and interobserver reliability of this novel method were evaluated. The results demonstrated that the ICCs of validity and intraobserver and interobserver reliability were all excellent for the four tested angles. One study suggested the use of 11 degrees to represent the 95% confidence interval for a measurement difference indicating a true change when measuring the Cobb angle in lateral spine films^15^. One study revealed that the minimum detectable change in T1S was 5.6 degrees^16^. The Bland‒Altman plot in this study showed that none of the results were beyond this acceptable limit. It is worth noting that the validity and intraobserver and interobserver reliability of NT were all lower than those of the other three angles. The possible reason is that the points (the sternum’s upper end and the centre of the T1 upper endplate) used to determine the connecting lines of NT were both illegible even on the selected radiographic films in this study. Additionally, our results revealed that this novel method is less time-consuming than the PACS method. Other advantages of this novel method include the solving of the problem of compatibility of radiographs from different hospitals; strong social mechanisms by which to share information and discuss clinical cases anytime and anywhere; no need to perform updates or make payments; and stability and quick response of the intrinsic function^10,14^.

The innovation of this method is not in changing the intrinsic measurement error of cervical sagittal parameter measurement but in providing a new, convenient measurement method^17^. In this study, the photos were taken on a vertical computer screen, and it is reasonable to speculate that photos taken with films located in a vertical film viewer machine could also use this novel measuring method. It is important to note that the radiographic film should be kept parallel to the camera when taking the photos in case of parallax error^18^. The radiographic films included in this study were legible in terms of anatomical structures such as the endplate of the vertebral body, the upper end of the sternum and the hard palate. However, in real clinical situations, these anatomical structures may be illegible, and the validity and reliability of the measurement may decrease.

## Conclusion

This novel method based on the iPhone’s intrinsic photo edit function is accurate, reliable, fast and convenient when measuring cervical sagittal parameters.

## Data Availability

The datasets used and/or analysed during the current study available from the corresponding author on reasonable request.
